# The Association of 25(OH)D with Blood Pressure, Pulse Pressure and Carotid-Radial Pulse Wave Velocity in African Women

**DOI:** 10.1371/journal.pone.0054554

**Published:** 2013-01-23

**Authors:** Iolanthé M. Kruger, Marlena C. Kruger, Colleen M. Doak, Aletta E. Schutte, Hugo W. Huisman, Johannes M. Van Rooyen, Rudolph Schutte, Leoné Malan, Nicolaas T. Malan, Carla M. T. Fourie, Annamarie Kruger

**Affiliations:** 1 Africa Unit for Transdisciplinary Health Research (AUTHeR), North-West University, Potchefstroom Campus, Potchefstroom, South Africa; 2 Institute of Food, Nutrition and Human Health, Massey University, Palmerston North, New Zealand; 3 Department of Nutrition and Health Institute of Health Sciences, Vrije University Amsterdam, Amsterdam, The Netherlands; 4 Hypertension in Africa Research Team (HART), North-West University, Potchefstroom Campus, Potchefstroom, South Africa; University of Perugia, Italy

## Abstract

High susceptibility of the African population to develop cardiovascular disease obliges us to investigate possible contributing risk factors. Our aim was to determine whether low 25(OH)D status is associated with increased blood pressure and carotid-radial pulse wave velocity in black South African women. We studied 291 urban women (mean age: 57.56±9.00 yrs.). 25(OH)D status was determined by serum 25(OH)D levels. Women were stratified into sufficient (>30 ng/ml), and insufficient/deficient (<30 ng/ml) groups. Cardiovascular variables were compared between groups. Women with low 25(OH)D levels had significantly higher SBP (150.8±27.1 vs. 137.6±21.0), DBP (94.7±14.5 vs. 89.3±12.3) and PP (53.15(50.7;55.7) vs. 46.3(29.4;84.6)) compared to women with sufficient levels. No significant difference was observed with regards to c-rPWV. ANCOVA analyses still revealed significant differences between the two groups with regards to SBP, DBP as well as PP. Partial correlations revealed significant inverse association between SBP and 25(OH)D (p = .04;r = −.12). Women with low 25(OH)D levels were ∼2 times more likely to have high SBP (95% CI: 3.23;1.05). To conclude, women with deficient/insufficient 25(OH)D had significantly higher SBP compared to women with a sufficient 25(OH) status.

## Introduction

For years 25(OH)D was associated primarily with bone mineralization and calcium homeostasis. However, over the past few years, ample literature has emerged indicating a myriad of bio-regulator effects of 25(OH)D on other physiological systems including the cardiovascular system [1], [2].

The African population is regarded as a high risk group for the development of cardiovascular diseases (CVD), especially hypertension and severe target-organ damage [3]. Numerous contributing factors involved in CVD have been investigated extensively to establish possible associations and even cause-and-effect. Despite considerable in-depth studies regarding CVD, it still remains one of the leading causes of morbidity and mortality.

The role of 25(OH)D deficiency in the development of CVD is receiving growing attention. Several studies have shown associations between low 25(OH)D levels and CVD [1], [2], more specifically through increased arterial stiffness [2], [4]. Most of these studies included only African-Americans or Caucasians and data regarding Africans from South Africa is scarce. This is an important gap in knowledge to address due to the high susceptibility of Africans for the development of hypertension. Thus, it is imperative to identify potential risk factors that contribute to cardiovascular dysfunction.

Our aim was to determine if a low 25(OH)D status is associated with increased blood pressure as well as increased arterial stiffness (muscular and elastic) in black South African women. The role of decreased aortic elasticity in cardiovascular disease is eminent; however, the clinical relevance of the resistance (muscular) arteries should not be neglected. Grey *et al* [5] emphasised the importance of reduced muscular artery elasticity, independent of age, in the prediction of cardiovascular event. According to Duprez *et al.*: “African-Americans are more prone than other groups to small artery disease than large artery disease” [6]. Furthermore, another study by Duprez *et al.* [7] demonstrated that systolic indices (augmentation pressure, augmentation index and systolic reflective index) derived from radial artery pressure waveform, are significantly and inversely related to small artery elasticity index.

According to a study performed by Tare *et al.* [8] 25(OH)D insufficiency is associated with impaired vascular endothelial and smooth muscle function. According to the National Health and Nutrition Examination Survey (NHANES) 42% of black women in the United States had 25(OH)D levels below 15 ng/ml [9]. The importance of this finding is further strengthened by the fact that 25(OH)D insufficiency/deficiency becomes more evident with age [10]. Furthermore, little is known about 25-hydroxyvitamin D in African women.

## Materials and Methods

### Ethics Statement

The study was approved by the Ethics Committee of the North-West University and complies with the Helsinki Declaration as revised in 2000. All the participants were fully informed about the study and the purpose in their preferred language. Participation was voluntary and they could withdraw at any time. All participants provided written consent.

### Research Design

This cross-sectional study was part of the South African leg of the Prospective Urban and Rural Epidemiology study (PURE) in the North West Province. The PURE study, coordinated from the Population Health Research Institute, Ontario, Canada, is a longitudinal study designed to track the development of chronic diseases of lifestyle, in urban and rural subjects, in approximately 20 developing countries [11]. Participating South African communities had to meet certain inclusion criteria. The main criteria were that communities had to show migration stability and also had to be part of the North-West Province of South Africa. Four different resident areas were identified for participation in the PURE-SA study. Community A was a rural community located ∼450 km west of Potchefstroom in the North-West Province of South Africa. Community B, a deep rural community 35 km east of A, was only accessible via a gravel road. Communities C and D were urban communities: C was an established township which forms part of the bigger Potchefstroom, whereas community D was informal settlements surrounding community C. Selected rural areas had to be far away from cities, still be under tribal law with as little urban influence as possible. With regards to the urban areas, the selected communities had to reflect a true representation of urbanization. Both urban and rural areas had to be big enough to make random selection of household possible.

### Subjects

Participants were voluntarily enrolled into the study (1 000 rural and 1 000 urban) from a population of 6 000 randomly selected households. Inclusion criteria for the volunteers in the PURE-SA study were a cohort of men and women older than 35 years who were not using medication for non-communicable diseases, not pregnant, were not intoxicated, and did not have any cognitive deficits. However, in the older age group participants using hypertension medication were included due to a very common use of chronic medications in this age cohort. For the purpose of this study, baseline data from all of the female participants aged >47 years and older from the urban group, collected over a 12 week period in 2005 were used. Participants who were HIV-infected were excluded from the analyses (Data from 3 HIV-infected participants were excluded from all analyses). Thus, the total group of women were 291.

### Measurements

For anthropometric measurements, the guidelines adopted at the National Institute of Health-sponsored Arlie Conference [12] were applied using standardised and calibrated apparatus. Measurements included weight (kg), height (meters), waist circumference (cm) and body mass index (kg/m^2^).

### Questionnaires

Eight field workers selected from the communities being researched administered all questionnaires in the preferred language of the participant. Fieldworkers were given extensive training to standardise the questionnaires. Data on alcohol consumption was obtained by means of a culturally sensitive quantitative food frequency questionnaire, developed and validated for use in this population [13]. Data on smoking habits were obtained by a questionnaire used and adopted for all countries participating in the PURE study. With regards to smoking habits and alcohol usage only dichotomous data (i.e. yes/no) were used.

### Daily Nutrient Intake

A culturally sensitive quantitative food frequency questionnaire, developed and validated for use in this population, was used to obtain the dietary (including alcohol) intake of the women. Portion sizes were estimated using a food portion photograph book developed for use in the African population in the North West Province. Portion sizes were reported in household measures and were concerted to weights using standard tables. The food intake was coded using the new food codes of the South African food composition database, developed by the Nutritional Intervention Research Unit of the South African Medical Research Council and expressed as average amounts consumed per day (http://sahealthinfo.org/nutrition/nutrition.htm).

### Blood Collection

Qualified nurses collected a fasting blood sample from the antecubital vein using a sterile winged infusion set and syringes. Samples for blood plasma were stored on ice until processing, whereas serum samples were allowed to clot at room temperature for 30 minutes. Collected plasma/serum samples were stored in aliquots in cryotubes at −80°C.

### Cardiovascular Measurements

Participants were seated in an upright and relaxed position. After a resting period of 10 minutes, systolic-, diastolic blood pressures and heart rate were measured using the Omron HEM-757 apparatus (Omron Healthcare, Kyoto, Japan). Measurements were taken in duplicate (5 min. apart) on the right arm (brachial artery) with the arm supported at heart level. Appropriate cuff sizes were used for participants.

Pulse pressure (PP) (a simple and readily obtainable correlate of elastic vessel stiffness) was determined. Pulse wave velocity of the muscular artery was measured using noninvasively accessible superficial pulses in an upper limb muscular artery over the carotid-radial segment (carotid-radial PWV (c-rPWV)) with the Complior SP device (Artech-Medical, Pantin, France). The c-rPWV was measured on the left side of each participant, while the subject was in a supine position.

### Biochemical Analysis

Sequential Multiple Analyzer Computer (SMAC), using the Konelab analyzer (Thermo Fisher Scientific Oy, Vantaa, Finland), was used for analyzing calcium, magnesium, albumin, γ-glutamyl transferase (GGT) and serum lipids. Low density lipoprotein cholesterol was calculated using the Friedewald formula [14]. Parathyroid hormone, 25(OH)D, and C-telopeptide of type I collagen (CTx) levels were measured using the Roche Elecsys 2010 COBAS system (Roche Diagnostics, Indianapolis, IN, USA). Plasma glucose was measured with a hexokinase method using the SynchronR System(s) (Beckman Coulter Co., Fullerton, CA, USA) and reagents. Serum creatinine was analyzed using the Cobas Integra 400 plus (Roche, Basel, Switzerland). The coefficients of variance (CV) for the above-mentioned assays were all <10%. Estimated creatinine clearance (eCcr) was calculated using the Cockcroft-Gault formula [15].

### HIV Testing

Written informed consent was obtained from each participant after pre-counseling before participation. Participants’ HIV status was determined using the First Response (PMC Medical, India) rapid HIV card test using whole blood. This test was performed according to the protocol of the National Department of Health of South Africa. If the First Response test was positive, it was confirmed with the Pareeshak card test (BHAT Bio-tech India). Feedback on results was given by two trained counselors during individual sessions just before the participants were transported back to their home. Infected participants were referred to their local clinic or hospital for follow-up and CD4 cell counts.

### Statistical Analysis

Data were analysed using the statistical software for social sciences (PASW Statistics 18 for Windows, SPSS Inc., Chicago, IL, USA). Data were checked for outliers and normality. Data that were not normally distributed were logarithmically transformed to follow an approximate normal distribution. Descriptive variables are presented as geometric mean (standard deviation)for normal distributed data and arrhythmic mean (5%;95% percentiles) for log-transformed data. Independent t-tests were performed to determine any significant differences between groups. One-way analyses of covariance (ANCOVA) were conducted to determine significant differences for variables whilst adjusting for potential confounders. Chi-square tests were used to determine significant differences between categorical variables. Forced entry multiple regression was performed in the total sample group to identify independent correlates of SBP, DBP, PP or c-rPWV. Preliminary analyses were conducted to ensure no violation of the assumptions of normality, linearity, multicollinearity and homoscedasticity. Binary logistic regression analyses were used to determine predictors for categorical dependent variables. *P*-values ≤.05 were considered significant. All *P*-values were two-sided hypotheses.

The group was stratified into two groups: Group 1– included women with 25(OH)D levels ≥30 ng/ml (sufficient levels) or Group 2 included women with levels below 30 ng/ml (insuficcient/deficient) [16].

## Results

Our study group had a mean age of 57.6±9.0 yrs. and circulating 25(OH)D levels of 26.0±1.4 ng/ml. Overall, the group had high SBP and DBP levels, with mean values of 145.9±25.8 mmHg and 92.7±13.9 mmHg respectively. The mean PP for the group was 50.5 mmHg and a c-rPWV of 10.3 m/s.

Descriptive characteristics are presented in Table 1. Women with low 25(OH)D levels revealed an unfavourable cardiovascular profile compared to participants with sufficient 25(OH)D levels. This is reflected by their significantly higher SBP, DBP and PP. No significant difference with regards to c-rPWV was observed between the two groups. Group 2 were also significantly older and had significant higher alcohol consumption.

**Table 1:** Descriptive statistics of the total sample and according to 25-hydroxyvitamin D status.

	Whole group	Group 1 (Sufficient) (>30 ng/ml)	Group 2 (Insufficient/deficient) (<30 ng/ml)
*n*	*291*	*106*	*184*
Age (yrs)	57.56 (9.00)	55.52(9.51) *	58.72 (8.50) *
Current smoking (%)	42.6	37.1	45.9
Current alcohol (%)	35.1	24.5*	41.1*
Blood pressure medication (%)	21.3	18.9	22.7
Body mass index (kg/m^2^)	27.59[17.75;41.60]	27.15 [18.43;38.98]	27.85 [26.74;29.01]
Waist circumference (cm)	83.95[64.88;107.25]	82.58 [65.30;102.45]	84.76 [82.66;86.90]
Waist-height ratio	53.73[40.98;68.49]	53.02 [40.87;66.11]	54.15 [52.84;55.48]
Vitamin D intake (µg/day)	2.36 [0.47;7.10]	2.34 [0.46;7.19]	2.38 [2.06;2.74]
*Cardiovascular measurements*			
Systolic blood pressure (mmHg)	145.92 (25.80)	137.58 (21.02) *	150.75 (27.11) *
Diastolic blood pressure (mmHg)	92.74 (13.94)	89.29 (12.30) *	94.73 (14.47) *
Pulse pressure (mmHg)	50.52[30.00;86.50]	46.28 [29.35;84.59] *	53.15 [50.69;55.73] *
Heart rate (bpm)	72.48 (15.04)	71.64 (13.01)	72.97 (16.11)
c-r PWV (m/sec)	10.26[7.09;14.32]	10.30 [6.97;14.70]	10.24 [9.95;10.53]
*Biochemical measurements*			
Fasting glucose (mmol/L)	5.05[3.40;8.10]	5.16 [3.42;9.52]	4.99 [4.81;5.18]
GGT (U/L)	51.42[18.60;278.80]	47.27 [17.31;251.71]	53.99 [48.14;60.55]
Creatinine (µmol/L)	85.90[42.61;378.97]	86.40 [44.35;378.97]	85.62 [77.21;94.97]
Creatinine clearance (ml/min)	66.31[13.79;160.32]	66.50 [15.42;141.09]	66.22 [59.27;73.98]
Parathyroid hormone (ng/L)	38.80[21.73;86.20]	37.49 [21.86;69.57]	39.57 [36.92;42.41]

c-rPWV: carotid-radial pulse wave velocity; GGT: gamma-glutamyl transferase; *n* number of participants.

Data expressed as arrhythmic mean (standard deviation) for normal distributed data and geometric mean [5%;95% percentile intervals] for log-transformed data or percentage of *n.*

* indicate significant difference between groups (p≤0.05).

All p-values for continuous variables were obtained by means of independent t-test and Chi-square for categorical variables.

After performing ANCOVA analyses (adjusting for age, BMI, alcohol consumption, smoking and blood pressure medication usage) the significant differences found for SBP (p = 0.004), DBP (p = 0.015) and PP (p = 0.023) remained (data not shown).

**Figure 1:**
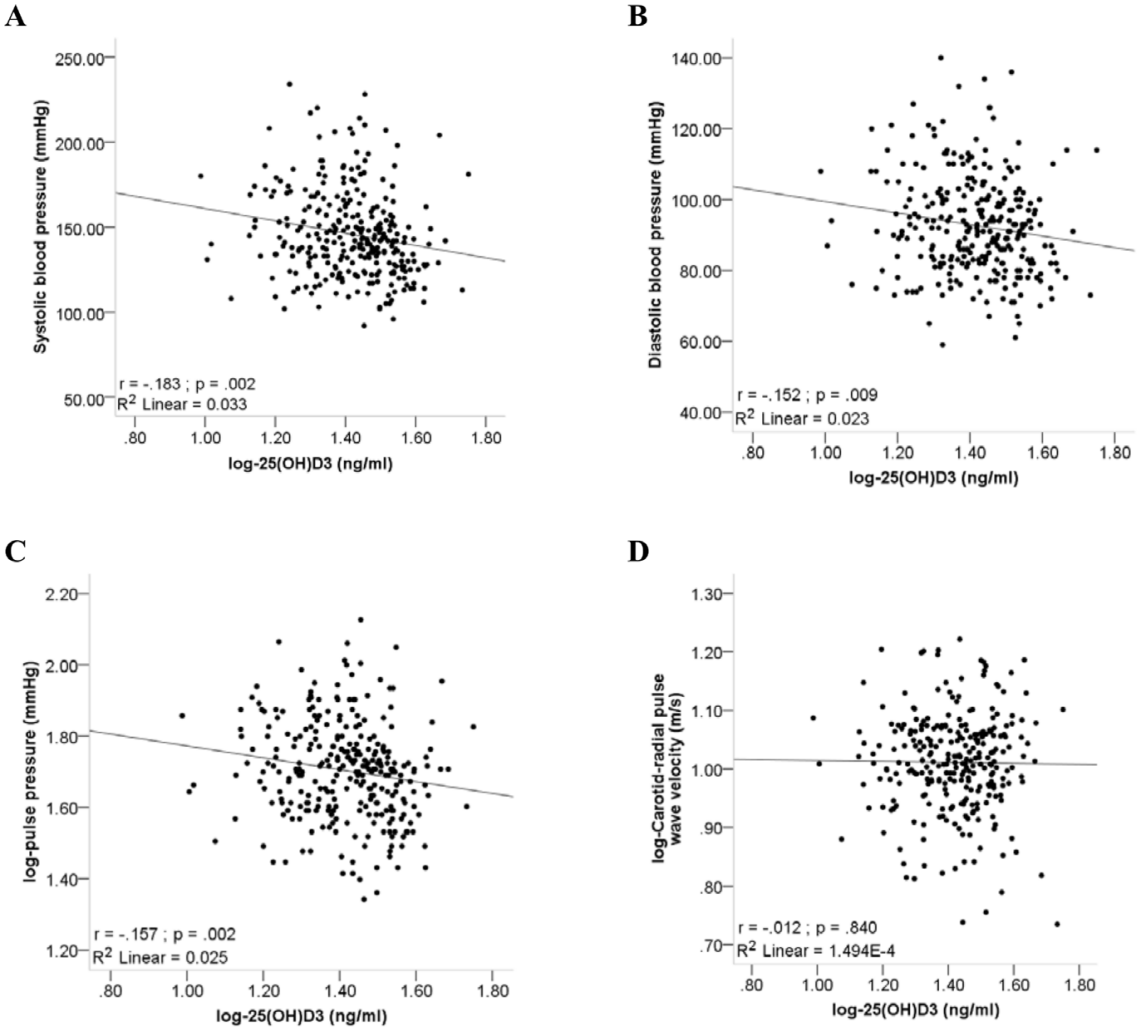
Single regression analysis between 25-hydroxyvitamin D levels and cardiovascular function. A) Systolic blood pressure, B) Diastolic blood pressure and C) Pulse pressure, D) Carotid-radial pulse wave velocity.

In order to determine whether low 25(OH)D levels are associated with poorer cardiovascular outcomes we plotted SBP, DBP, PP and c-rPWV against 25(OH)D levels (Fig. 1).

Figure 1 illustrates significant inverse correlations between blood pressure measures (SBP and DBP) and 25(OH)D levels (N = 291; r =  −.183 and r =  −.152 respectively). Even though the associations were significant, they were relatively weak. A significant inverse association between 25(OH)D and PP was observed (r =  −.157; p = .025), yet no association was observed between c-rPWV and 25(OH)D levels (r = .012; p = .840).

Partial correlation analyses were used to further explore the relationship between SBP, DBP, PP and c-rPWV with 25(OH)D whilst adjusting for potential confounders (age, BMI, smoke, alcohol and blood pressure medication). Only the significant inverse association found between SBP and 25(OH)D remained (p = .04; r =  −.12).

Multiple regression analyses with either SBP, DBP, PP or c-rPWV as dependent variable were performed to confirm the results found from the partial correlations (Table 2). The effect of age was strongest on SBP (β = .267; p<.001), whereas BMI had the greatest independent effect on DBP (β = .254; p<.001). The effect of age (β = .414; p<.001) and mean arterial pressure (β = .208; p = .001) seem to be the most significant on PP and c-rPWV respectively. Furthermore, 25(OH)D levels seems to play a significant role in the variance of SBP (β = -.125; p = .035), whereas the variance in none of the other dependent variables seem to be influenced by 25(OH)D levels.

**Table 2:** Multiple regression analysis for blood pressure and arterial stiffness.

Regressors	Systolic blood pressure			Diastolic blood pressure			Pulse pressure			Pulse wave velocity		
*R^2^ adjusted*	0.102			.079			.174			.089		
	β	Partial *R^2^*	*P*	β	Partial *R^2^*	*P*	β	Partial *R^2^*	*P*	β	Partial *R^2^*	*P*
Age	**.267**	**.062**	**<.001**	−.056	.003	*NS*	**.414**	**.147**	**<.001**	**.145**	**.017**	**.027**
BMI (kg/m^2^)	.119	.001	*NS*	**.254**	**.055**	**<.001**	−.048	.002	*NS*	–	–	–
Height (m)	–	–	–	–	–	–	–	–	–	.075	.005	*NS*
MAP (mmHg)	–	–	–	–	–	–	–	–	–	**.208**	**.040**	**.001**
HR (bpm)	–	–	–	–	–	–	–	–	–	.092	.008	*NS*
25(OH)D (ng/mL)	**−.125**	**.014**	**.035**	−.110	.011	*NS*	−.101	.009	*NS*	.096	.008	*NS*
Smoking	−.071	.004	*NS*	.007	<.001	*NS*	−.104	.008	*NS*	.088	.006	*NS*
Alcohol use	**.171**	**.020**	**.012**	**.210**	**.030**	**.002**	.088	.005	*NS*	**.143**	**.014**	**.040**
PTH	−.023	.001	*NS*	.031	.001	*NS*	−.009	<.001	*NS*	−.028	.001	*NS*

BMI: body mass index; MAP: mean arterial pressure; HR: heart rate; 25(OH)D: 25-hydroxyvitamin D.

Values in bold revealed the strongest associations with the dependent variables.

*NS* not significant.

Additional analysis using a binary logistic regression showed that 25(OH)D status is a significant predictor (p = .032) for high SBP. The odds of women that are 25(OH)D insufficient/deficient are 1.85 times higher [95% CI: 3.23;1.05] to have high SBP compared to women that have sufficient 25(OH)D levels.

## Discussion

Our aim was to determine if a low 25(OH)D status is associated with increased blood pressure as well as increased muscular- and elastic arterial stiffness (reflected as carotid-radial pulse wave velocity and pulse pressure respectively) in South African women. One of the main findings from this study showed that women with low 25(OH)D levels had significantly higher systolic blood pressure compared to women with sufficient levels of 25(OH)D, they also had ∼2 times higher odds of having high SBP compared to women with a sufficient status.

African-Americans are known to have lower blood concentrations of 25(OH)D compared to their European-American counterparts [17]. According to a previous South African study, low levels of circulating 25(OH)D was also observed in black adult African population [18]. The reasons are multifactorial and include genetics [19], low 25(OH)D intake [20], higher prevalence of obesity [21] and another important factor: dark pigmented skin. Due to the presence of high levels of epidermal melanin in the skin it reduces the amount of ultraviolet entering the skin (photo-protective characteristic) thereby inhibiting the production of 25(OH)D_3_ [22]. Data from our study corresponds well with the literature with regards to deficient 25(OH)D levels. An alarming 63.6% of the women had levels below the 30 ng/ml optimum cut-point.

The inverse relationship between systolic blood pressure and 25(OH)D levels observed in our study is in accordance with previous studies [8], [23]. The link with the cardiovascular system may be explained by indirect but also direct effects of 25(OH)D on the heart and vasculature. 25(OH)D mediates its effects via ligand-regulated vitamin D receptors [24] expressed in target organs directly involved in calcium homeostasis (bones, kidneys, intestinal tract, and the parathyroid gland), as well as other target organs including skeletal muscles, smooth muscle cells, and myocardium [25]. Through selective activation of vitamin D receptors, 25(OH)D exerts hormonal, autocrine and paracrine actions [26] that are increasingly recognized as essential to maintaining cardiovascular health [27].

A potential mechanism linking 25(OH)D directly in the regulation of blood pressure is put forward by Li *et al* [28]. The authors demonstrated that 1,25(OH)_2_D_3_ functions as a negative endocrine regulator on the renin-angiotensin system (RAS). They discovered that knock-out mice missing the vitamin D receptor had increased renin mRNA in the kidney as well as increased plasma Angiotensin II production. As a consequence of over stimulation of the RAS, these mice developed hypertension. In addition, they also found that renin up-regulation was evident prior to hypocalcaemia suggesting that regulation of renin by 1,25(OH)_2_D_3_ is independent of serum calcium. However, it is well known that the African population, especially African women, have low plasma renin activity [29], therefore, the latter mentioned is not a plausible explanation. A possible indirect mechanism linking increased blood pressure with low 25(OH)D levels is put forward by Kriebitzsch et al [30], [31]. The authors propose that 1,25-dihydroxyvitamin D_3_ induces a rapid transcription of a cluster of genes including the cystathionine beta synthase (*cbs*) gene, which encodes the key enzyme in the trans-sulfuration pathway. The cbs enzyme is a trans-sulfuration enzyme which is required for the irreversible catabolism of homocysteine [32]. Consequently, deficient levels of 25(OH)D will result in low 1,25-dihydroxyvitamin D_3_ levels with a possible concomitant rise in homocysteine, a known hypertension risk factor [33].

Previous studies revealed a negative relationship between 25(OH)D status and elastic artery stiffness [2]. We have demonstrated that with a decrease in 25(OH)D levels there is a concomitant increase in elastic artery stiffness, measured by PP. However, after adjusting for potential confounders, this significant association disappeared. The reason may be due to the fact that increased PP may be both a causal factor for CVD [34] but also a consequence of atherosclerosis [35]. 25(OH)D is known for its protective properties against artery calcification [36], perhaps it can be speculated that low 25(OH)D levels may cause an indirect increase in PP via artery calcification. Conceivably the degree of arterial calcification was not severe in our group, thus a significant result was not obtained. In contrast to findings from a recent study by Pirro et al [37], PTH seems not to be a significant confounder for the association between 25(OH)D and elastic artery in our study group. This discrepancy might be explained by the different measures for elastic artery stiffness (pulse pressure vs. aortic PWV), different threshold values for 25(OH)D (20 ng/ml vs. 30 gn/ml) as well as age difference (our group was 10 yrs. younger).

Previously, most studies only investigated the association between 25(OH)D and *elastic* artery stiffness. Our study also included an indicator of *muscular* artery stiffness (carotid-radial pulse wave velocity). However, our findings are not consistent with the findings of Tare *et al* [8]. In our population, we found no association between low 25(OH)D status and increased muscular artery stiffness. When a standard multiple regression analysis was performed, it confirmed the fact that 25(OH)D levels do not play a significant role and that c-rPWV is more strongly influenced by mean arterial pressure. This observation is in accordance with an earlier article published by Schutte *et al.* [38] who also found increased muscular arterial stiffness (PWV) to be strongly associated with blood pressure in a black South African population. There are some potential limitations to this study. Firstly, our study is limited by its cross-sectional design, which prevents us from drawing any conclusion on causality. Secondly, the assay used to measure 25(OH)D only measured 25(OH)D_3_ and not 25(OH)D_2_, thus the total 25(OH)D is not known. Furthermore, renin and angiotensin II (indicators of RAS activity) and markers for liver function were not measured. Another limitation is the lack of other markers of elastic artery stiffness, such as the augmentation index and carotid-femoral PWV, which is considered to be the golden standard for large artery stiffness. Lactation status and lactose intolerance were also unknown. Despite these limitations, the results from this study are still very relevant and novel. It is further recommended that follow-up studies be performed to include both older as well as younger participants. According to our knowledge no other studies investigated the association of 25(OH)D with blood pressure and arterial stiffness within a black South African population.

The translational potential of knowledge gained from the current and potential future projects may help in preventative programmes for individuals who are at high risk for hypertension. Some individuals are known to have some degree of genetic load for blood pressure [39]. A rise in blood pressure starts at a much earlier age in Africans compared to their Caucasian counterparts Schutte *et al* [38]. It is difficult to determine whether the relationship between 25(OH)D and blood pressure found here is driven by inheritance or by lifestyle and environment. According to a set of guidelines from the IMAGE study group for the prevention of type 2 diabetes [40], an important approach to preventative therapies/programmes is “small changes in lifestyle will bring big changes in health”. An unfavourable 25(OH)D status at an early age might exacerbate predisposed hypertension. Thus effective prevention of 25(OH)D deficiency and related health risk through appropriate awareness and preventative programmes might help to diminish the high prevalence rates of hypertension found in black South Africans.

In conclusion, our findings indicate that African women with insufficient/deficient 25(OH)D levels are ∼2 times more likely to have high blood pressure compared to women with normal levels. There is an urgent need to clarify causality with randomized clinical trials. These results show a serious health problem with a potential for wide-scale intervention such as vitamin D fortification and supplementation appropriate to reaching this population.
